# Cellular Attachment and Differentiation on Titania Nanotubes Exposed to Air- or Nitrogen-Based Non-Thermal Atmospheric Pressure Plasma

**DOI:** 10.1371/journal.pone.0113477

**Published:** 2014-11-24

**Authors:** Hye Yeon Seo, Jae-Sung Kwon, Yu-Ri Choi, Kwang-Mahn Kim, Eun Ha Choi, Kyoung-Nam Kim

**Affiliations:** 1 Department and Research Institute of Dental Biomaterials and Bioengineering, Yonsei University College of Dentistry, Seoul, Republic of Korea; 2 BK 21 Plus Project, Yonsei University College of Dentistry, Seoul, Republic of Korea; 3 Plasma Bioscience Research Center, Kwangwoon University, Seoul, Korea; University Paul Sabatier, France

## Abstract

The surface topography and chemistry of titanium implants are important factors for successful osseointegration. However, chemical modification of an implant surface using currently available methods often results in the disruption of topographical features and the loss of beneficial effects during the shelf life of the implant. Therefore, the aim of this study was to apply the recently highlighted portable non-thermal atmospheric pressure plasma jet (NTAPPJ), elicited from one of two different gas sources (nitrogen and air), to TiO_2_ nanotube surfaces to further improve their osteogenic properties while preserving the topographical morphology. The surface treatment was performed before implantation to avoid age-related decay. The surface chemistry and morphology of the TiO_2_ nanotube surfaces before and after the NTAPPJ treatment were determined using a field-emission scanning electron microscope, a surface profiler, a contact angle goniometer, and an X-ray photoelectron spectroscope. The MC3T3-E1 cell viability, attachment and morphology were confirmed using calcein AM and ethidium homodimer-1 staining, and analysis of gene expression using rat mesenchymal stem cells was performed using a real-time reverse-transcription polymerase chain reaction. The results indicated that both portable nitrogen- and air-based NTAPPJ could be used on TiO_2_ nanotube surfaces easily and without topographical disruption. NTAPPJ resulted in a significant increase in the hydrophilicity of the surfaces as well as changes in the surface chemistry, which consequently increased the cell viability, attachment and differentiation compared with the control samples. The nitrogen-based NTAPPJ treatment group exhibited a higher osteogenic gene expression level than the air-based NTAPPJ treatment group due to the lower atomic percentage of carbon on the surface that resulted from treatment. It was concluded that NTAPPJ treatment of TiO_2_ nanotube surfaces results in an increase in cellular activity. Furthermore, it was demonstrated that this treatment leads to improved osseointegration *in vitro*.

## Introduction

Titanium (Ti) is commonly used for medical and dental implants, and the surface properties of Ti implants, including the chemical composition, energy levels, morphology, topography and roughness, have been widely studied [1]. Among these factors, the surface topography and chemistry of Ti implants are known to be important for successful osseointegration [2], [3], though the results of previously reported studies were influenced by some parts of the experimental design, including sample preparation method, sterilization and cell type, as shown by Faghini et al. [4], Wirth et al. [5], and Chien et al. [6]. The implant surface topography is known to be the main influencing factor on osteoblastic cell adhesion and fibroblast responses [7], [8], [9], and topographical modifications of the implants surfaces have been attempted to improve the integration of hard and soft tissues [10]. Recently, surfaces composed of nanostructures such as titania (TiO_2_) nanotubes have been studied because the topographical modifications using these structures were reported to be superior in promoting early biological events related to the adsorption of proteins, blood clot formation and cell behavior [11], which all resulted in a higher degree of osseointegration [12].

However, certain cell bioactivity is more affected by the surface chemistry than by the surface topography [7], as observed in recently developed, highly hydrophilic implant surfaces with better osseointegration than conventional surfaces [13]. The effects of plasma on Ti surfaces have been widely studied, in particular regarding the modification of surface roughness, wettability and cell-surface interactions that result from treatment [14], [15], [16]. Despite many attempts to modify the chemical features of Ti surfaces, the time-dependent degradation of the chemical effects is substantial in that the strength of osseointegration is reduced in aged Ti surfaces compared with newly prepared Ti surfaces [17]. Additionally, chemical modifications made in a high-energy vacuum environment often result in the disruption of beneficial topographical features on biomaterials [18].

Non-thermal atmospheric pressure plasma has been recently applied in many biological fields because it has the advantage of being sufficiently low in temperature that it can be used on biomaterial surfaces without resulting in topographical changes [19]. Additionally, the atmospheric pressure plasma device does not require a vacuum environment to operate [20], making it portable and thus usable for the treatment of biomaterial surfaces immediately before their application. The plasma treatment of biomaterials is known to render surfaces hydrophilic and to modify the oxide layer that interacts with the proteins and cells of surrounding tissue, which leads to an increased adhesion of cells and tissue compared with samples not treated by plasma [20], [21], [22]. One of the most popular non-thermal atmospheric pressure plasma sources is based on the dielectric barrier discharge, which has previously been applied to biomaterials in many studies [23]. However, there has not yet been a study that has applied an NTAPPJ to TiO_2_ nanotubes.

Therefore, this study was performed with the purposes of applying a non-thermal atmospheric pressure plasma jet (NTAPPJ) produced using one of two gas sources to the surfaces of nanotube-based dental implants directly before surgery and of determining whether this procedure improved their hydrophilicity and osteogenic properties. It was hoped that this procedure would prevent the loss of the benefits of chemical modification while preserving the topographical morphology.

## Materials and Methods

### Preparation of TiO_2_ Nanotubes

Commercially pure Ti discs (grade IV, 10×10×0.4 mm) were polished with #400, #600, #800, #1200 and #2000 grit SiC sandpaper and were then ultrasonically cleaned with acetone, alcohol and distilled water, for 15 min each. The TiO_2_ nanotubes were formed by electrochemical anodization using a direct current power supply (Genesys, 600-2.6 Densei-Lambda Tokyo, Japan), during which a constant voltage of 20 V was applied for 1 h with an electrolyte solution containing 0.1 wt% hydrofluoric acid (HF). The samples were then dried at room temperature for 24 h and annealed at 450°C. All the specimens were sterilized using ethylene oxide (EO) gas at a temperature of 55°C for 1 h before each experiment. EO gas sterilization was chosen for sterilization of TiO_2_ nanotubes in the cell experiments because previous experiments we have verified that it has no effect on cell health [24], [25].

### NTAPPJ Treatment

An NTAPPJ device was manufactured from the Plasma Bioscience Research Center (PBRC, Kwangwoon University, Korea); details concerning the device design can be found in a previous study [26]. Briefly, the working gas (either compressed air or nitrogen) was used at a gas flow of 5 L/min, and the distance between the NTAPPJ tip and TiO_2_ nanotube surface was set to 3 mm. Quartz with a depth of 3.2 mm was used as the dielectric and stainless steel was used as the outer electrode, which enclosed porous alumina of 150 to 200 µm pore size and 35% porosity. The total output power of the plasma was set to 2.4 W for both the nitrogen and air NTAPPJs. The discharges were formed at a discharge voltage of 2.24 kV and a discharge current of 1.08 mA. The discharge duration was 0.18 ms and the discharge filamentation frequency was 12 kHz during a discharge voltage period of 16 ms, which corresponds to a discharge frequency of 60 Hz. The number of discharges within the 1-ms discharge voltage period was approximately 100.

Three different NTAPPJ treatment times (0, 2 and 10 min) were used for the experiments. The groups subjected to these treatment times were labeled as NP0, NP2 and NP10 for nitrogen gas and as AP0, AP2 and AP10 for compressed air. NP0 and AP0 were used as controls.

### Surface Characterization

The morphologies of the TiO_2_ nanotube surfaces before and after the 10 min NTAPPJ treatment were examined using a field-emission scanning electron microscope (FE-SEM, JSM-7100F, JEOL, Japan) and an optical 3D surface profiler (ContouGT, Bruker, USA). An optical profiler is device that measures the surface height optically and represents the height as a color on a scale ranging from red to blue, on either 2D or 3D images of the surface. The optical profilometer was thus easily used for evaluation of the surface roughness difference at a particular position before and after treatment. The surface roughness characteristics were analyzed using the vertical scanning interferometry mode (VSI) using a green luminous source. Quantification of the roughness parameters was performed using software at a magnification of 10× with a scanning area of 63 µm×47 µm.

The wettability of both the test and control samples was measured by dropping 4 µL of distilled water on the sample and then measuring the contact angle after 10 s using a video contact angle goniometer (Phoenix 300, SEO, Korea).

Finally, the chemical compositions of the test and control sample surfaces were determined using an X-ray photoelectron spectroscope (XPS, K-alpha, Thermo VG, UK). Photoelectrons were generated by a monochromatic Al Kα line (1486.6 eV) X-ray source, and the beam was powered at 12 kV and 3 mA, at a beam diameter of 400 µm. The Ti2p, O1s and C1s peaks were analyzed for chemical property changes. The binding energy was calibrated using the C1s at the 284.8 eV peak.

### Cell Culture

The MC3T3-E1 murine pre-osteoblast cell line (CRL-2593, American Type Culture Collection, USA) was used for the cell attachment/viability test. The cells were cultured in alpha-MEM cell culture medium (LM008-01, Welgene, Korea) combined with 10% fetal bovine serum (FBS, Gibco, USA), penicillin (100 units/ml, Gibco, USA) and streptomycin (100 mg/ml, Gibco, USA) at 37°C in 5% CO_2_. The cell culture media were changed every 48 h. For the gene expression analysis test, rat mesenchymal stem cells (rMSC, Lonza, USA) were used. The cells were cultured in alpha-MEM supplemented with 10% FBS and the same amount of penicillin/streptomycin as above. Cells from passages 3 to 5 were used in this study.

### Cell Attachment and Viability

For the cell attachment and viability tests, 1×10^5^ cells of MC3T3-E1 were placed on each of the test and control samples; after 4 h for attachment, the surfaces of the samples were washed using Dulbecco's phosphate-buffered saline (DPBS, Gibco, USA) to remove any non-adherent cells. Calcein AM and ethidium homodimer-1 stains (LIVE/DEAD Viability/Cytotoxicity Kit, Invitrogen Co., Eugene, Oregon, USA) were then applied on each sample, and the levels of staining were assessed using a confocal laser microscope (CLSM 700, Carl Zeiss, Jena, Germany) to evaluate cell attachment and cell viability. The numbers of cells attached to the test and control surfaces were also assessed by water soluble tetrazolium (WST, Dae-il Lab, Seoul, Korea) assay. This assay was performed after 4 h, 24 h, 3 d and 5 d of culture, and the results are expressed as the percentage to the control sample.

### Gene Expression Analysis

Real-time reverse transcriptase-polymerase chain reaction (RT-PCR) was used to determine the mRNA expression levels of osteogenic genes for rMSCs cultured on the NTAPPJ-treated and NTAPPJ-untreated specimens. RT-PCR was used to analyze, in particular, the expression levels of alkaline phosphatase activity (ALP), runt-related transcription factor 2 (Runx2), osteopontin (OPN), osteocalcin (OCN) and the house-keeping gene glyceraldehyde 3-phosphate dehydrogenase (GAPDH). Following plasma treatment, 1×10^4^ rMSCs were placed on both the control and test samples and were incubated at 37°C in 5% CO_2_, for either 7 or 14 days. The total RNA was then isolated from the cells with Trizol reagent (Sigma-Aldrich Company, St. Louis, MO, USA), and the RNA was converted into cDNA using an Omniscript RT kit (Qiagen, Hilden, Germany), which was incubated at 37°C for 90 min. Data analysis was performed using an ABI Prism 7500 machine (Applied Biosystems, Foster City, CA, USA). The conditions for PCR were as follows: a 95°C/10 minute activation step, followed by a denaturation step of 95°C/15 seconds and a primer extension of 60°C/minute for 40 cycles. The gene expression data were normalized with GAPDH expression, and the results are expressed as the relative fold increase in gene expression compared with the control, i.e. TiO_2_ nanotubes not treated with NTAPPJ.

### Statistical Analysis

All of the cellular experiments were performed 3 times using 4 samples of each test and control group. To identify any significant differences between the groups, the data were subjected to a one-way analysis of variance (ANOVA) and Tukey's test. Significance was determined at the 95% confidence level.

## Results

### Surface Characterization

**Figure 1:**
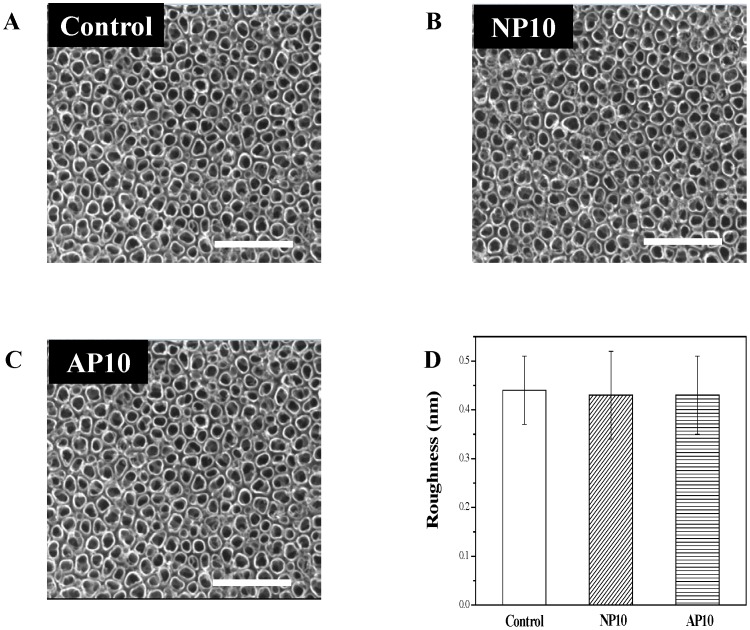
FE-SEM micrographs of the Ti specimens and the average roughness (Ra) values of the TiO_2_ nanotubes. (A) untreated TiO_2_ nanotubes (control), (B) TiO_2_ nanotubes with 10-min nitrogen-based NTAPPJ treatment, (C) TiO_2_ nanotubes with 10-min air-based NTAPPJ treatment, (D) average roughness (Ra) values for control and test TiO_2_ nanotubes. Scale Bar: 500 µm.

Fig. 1 shows FE-SEM images of the morphology of the TiO_2_ nanotube surface before (Fig. 1A) and after (Figs. 1B and 1C) the NTAPPJ treatment, as well as the average surface roughness (Ra) values determined using a surface profiler (Fig. 1D). The results indicate that there was no change in the topographic characteristics of the TiO_2_ nanotube surfaces in terms of either the morphology or average roughness values.

Contact angle measurements are listed in Table 1. The water contact angle of the TiO_2_ nanotube surface was significantly reduced following NTAPPJ treatment (*P*<0.05); in particular, both the NP2 and NP10 groups are ultra-hydrophilic and have contact angles of 0°. Although there was a significant decrease in contact angle for the AP2 and AP10 groups, their contact angles were reduced less compared with the control group than those of NP2 and NP10.

**Table 1:** The water contact angle (°) of the control and test TiO_2_ nanotube surfaces at room temperature.

Samples	Mean ± standard deviation contact angle (°)
Control group	46.4±4.0
NP2	0.0^*^
NP10	0.0^*^
AP2	26.38±4.43^*^
AP10	33.81±5.10^*^

NP2: TiO_2_ nanotube with 2-min nitrogen-based NTAPPJ; NP10: TiO_2_ nanotube with 10-min nitrogen-based NTAPPJ; AP2: TiO_2_ nanotube with 2-min air-based NTAPPJ; AP10: TiO_2_ nanotube with 10-min air-based NTAPPJ.

The symbol ‘^*^’ indicates a significant difference compared with the control group when analyzed using one-way ANOVA (*p*<0.05).

**Figure 2:**
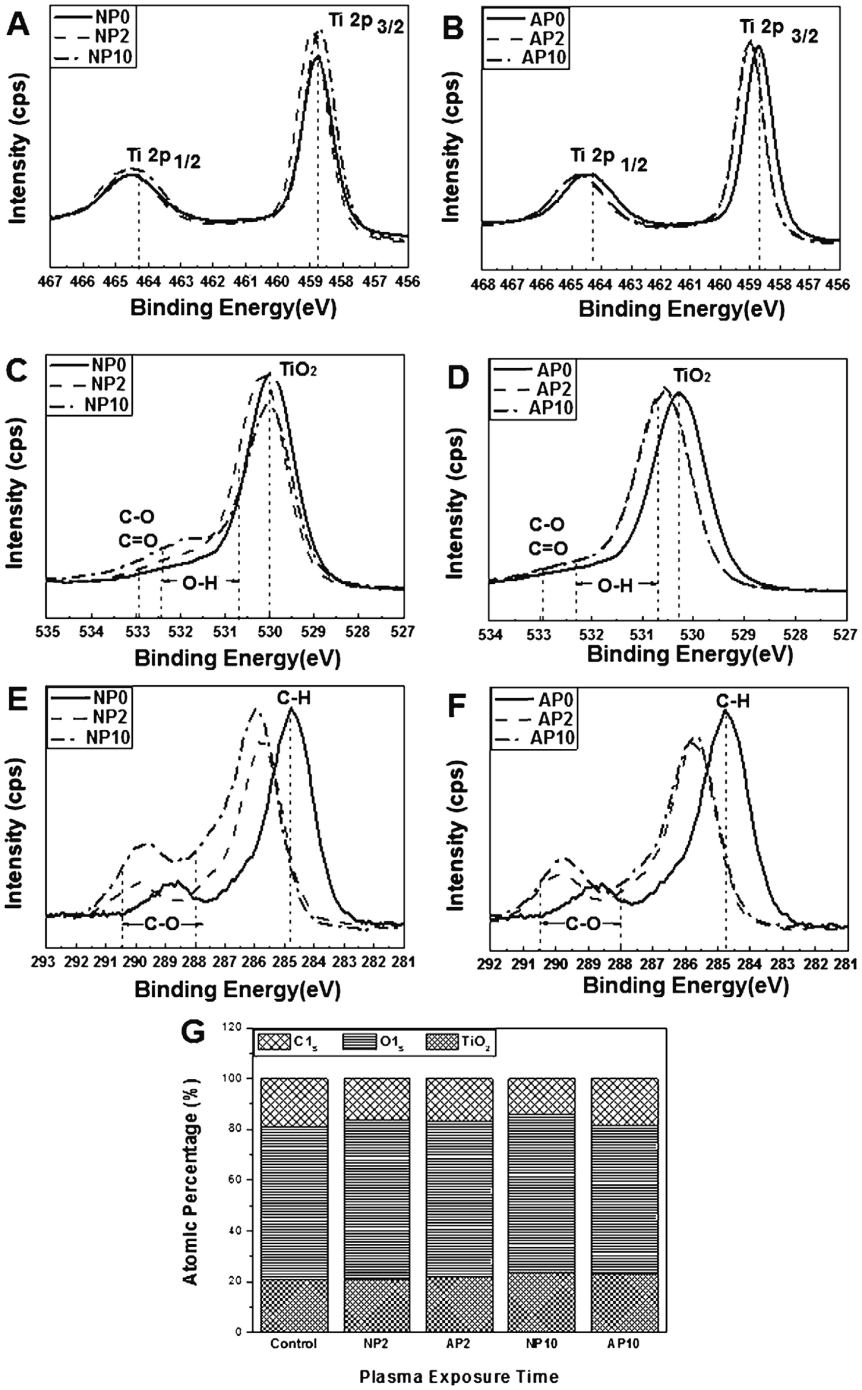
Chemical composition of a TiO_2_ nanotube surface measured with XPS. (A, B) Ti2p spectra, (C, D) O1s spectra and (E, F) C1s spectra. (G) Atomic percentage of each element on the surface of TiO_2_ nanotubes before and after nitrogen- or air-based NTAPPJ treatment. NP0 and AP0: untreated TiO_2_ nanotubes with no treatment (control group); NP2: TiO_2_ nanotubes with 2-min nitrogen-based NTAPPJ; NP10: TiO_2_ nanotubes with 10-min nitrogen-based NTAPPJ; AP2: TiO_2_ nanotubes with 2-min air-based NTAPPJ; AP10: TiO_2_ nanotubes with 10-min air-based NTAPPJ.

The XPS spectra revealed the peaks for Ti2p, O1s and C1s. In Figs. 2A and 2B, a Ti2p doublet peak is visible, containing both Ti2p 1/2 and Ti 2p 3/2 components and appearing from 464.3 eV to 458.7 eV (difference of 5.6 eV). The O1s spectra, presented in Figs. 2C and 2D, contain a peak corresponding to TiO_2_ near 530.0 eV; the presence of this peak was attributed to the lattice oxygen in the sample [27]. A peak is also visible in each from 532.4 eV to 530.7 eV that corresponds to the hydroxyl groups (O–H) [28], [29]. The peak at 532.9 eV is associated with C-O and/or C = O bonds [27]. The Ti2p and O1s peaks were slightly shifted to higher binding energies in the experimental groups compared with the control group.

In the case of the C1s peaks (Figs. 2E and 2F), the peak corresponding to hydrocarbon (284.7 eV) was decreased in the experimental groups. Additionally, the shoulder peak between 290.5 eV and 288.0 eV(C-O) was significantly shifted to a higher binding energy for all of the experimental groups compared with the control group [30], [31].

The atomic percentage of each element in the samples was analyzed from the XPS peaks (Fig. 2G). The carbon content of the experimental groups was lower than that of the control group (19.0%). The nitrogen-based NTAPPJ treatment group exhibited a lower carbon content (NP2 15.9%, NP10 13.8%) than the air-based NTAPPJ treatment group (AP2 16.8%, AP10 18.2%) for the same duration of exposure.

### Cell Attachment and Viability

**Figure 3:**
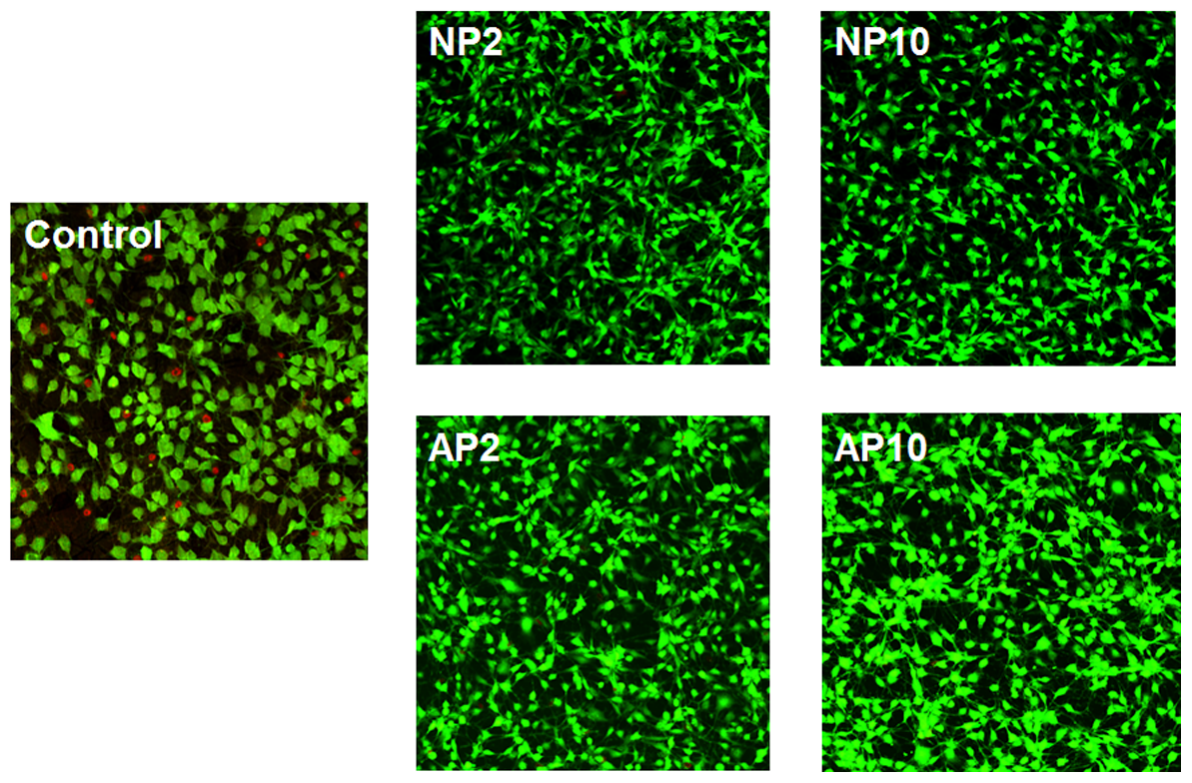
Cell attachment and viability on TiO_2_ nanotubes before and after the NTAPPJ treatment. Live cells are green, and dead cells are red. Control: untreated TiO_2_ nanotubes with no treatment; NP2: TiO_2_ nanotubes with 2-min nitrogen-based NTAPPJ; NP10: TiO_2_ nanotubes with 10-min nitrogen-based NTAPPJ; AP2: TiO_2_ nanotubes with 2-min air-based NTAPPJ; AP10: TiO_2_ nanotubes with 10-min air-based NTAPPJ.

The cells were stained with calcein AM and ethidium homodimer-1 to determine both the number of cells attached to a sample and the viability of the cells, as indicated by the green (live cells) or red (dead cells) colors in images (Fig. 3). The smallest numbers of cells were attached to the substrates in the control group, and some cells appeared to be dead on these samples. In contrast, all of the cells on the test samples were green (indicating live cells), and a relatively large number of cells were attached. Additionally, the cells were rounder in shape for the control group, whereas cells had stretched shapes in the experimental group. In particular, the cells on NP2 and NP10 contained more stretched filopodia than the cells in the control groups, AP2 and AP10.

**Figure 4:**
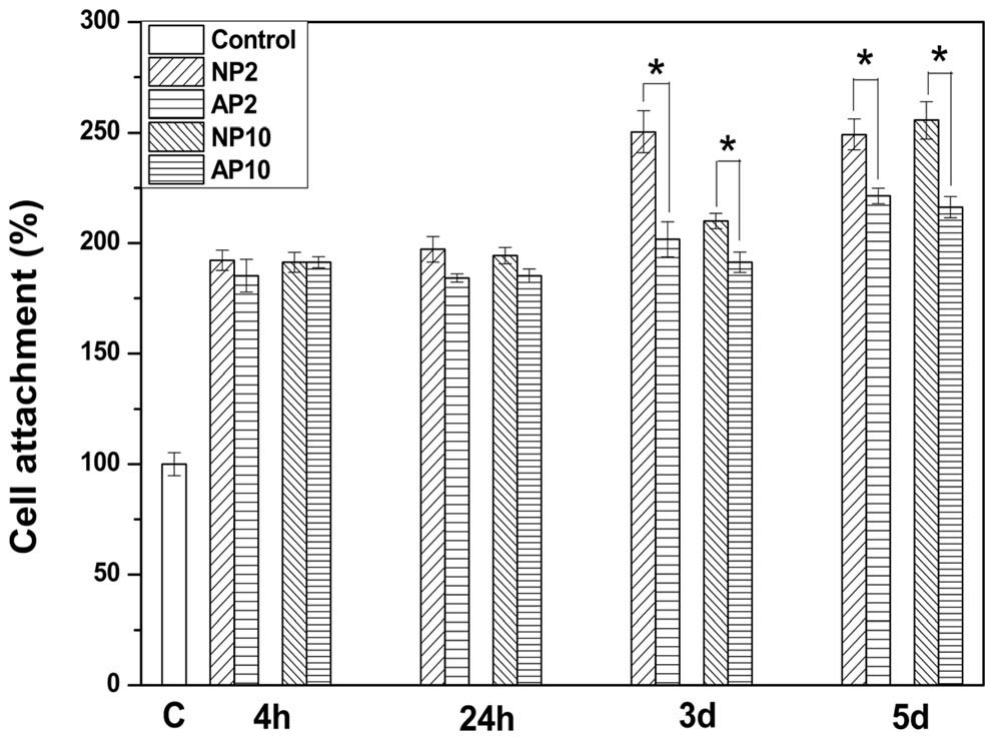
Numbers of cells attached on the surface of TiO_2_ nanotubes before and after the NTAPPJ treatment. C: untreated TiO_2_ nanotubes with no treatment; NP2: TiO_2_ nanotubes with 2-min nitrogen-based NTAPPJ; NP10: TiO_2_ nanotubes with 10-min nitrogen-based NTAPPJ; AP2: TiO_2_ nanotubes with 2-min air-based NTAPPJ; AP10: TiO_2_ nanotubes with 10-min air-based NTAPPJ. Significant differences in numbers of cell attachments between NTAPPJ with different gas sources for the same duration of exposure on TiO_2_ nanotubes are marked with ‘*’ (p<0.05).

The numbers of cells attached to each of the control and test samples are presented in Fig. 4. From these results, it is apparent that there was significant increase (p<0.05) in the numbers of attached cells following exposure to either air or nitrogen-based NTAPPJ compared to the control group, for any exposure time. Furthermore, in both days 3 and 5 of cell culture, there was a significant difference in the numbers of cells that attached onto samples treated with air-based NTAPPJs and those that attached onto samples treated with nitrogen-based NTAPPJs, for each of duration of exposure. In all cases, nitrogen-based NTAPPJ-treated TiO_2_ nanotubes displayed better cell attachment (p<0.05).

### Gene Expression Analysis

**Figure 5:**
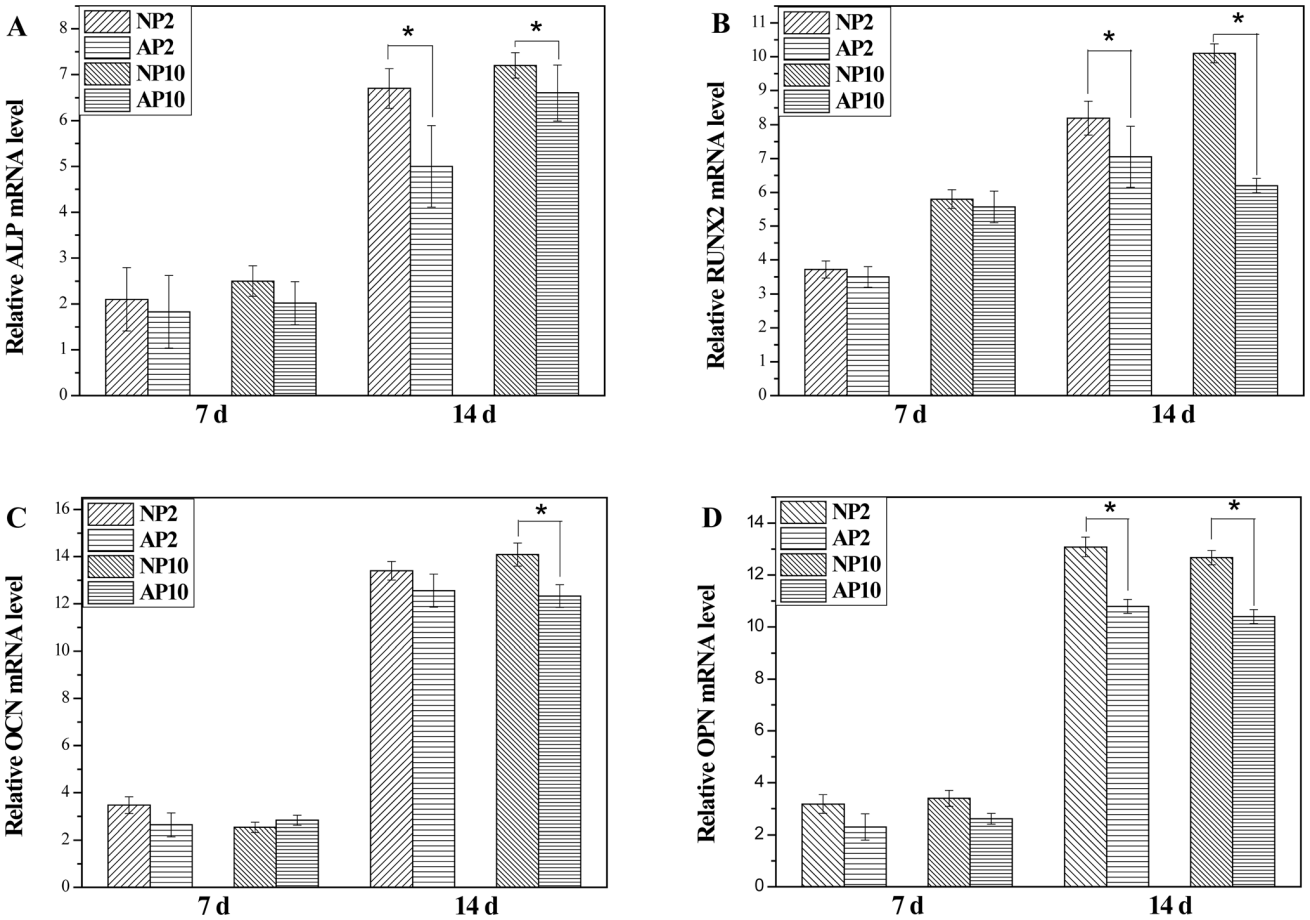
Real-time PCR results for relative osteogenic gene expression of rMSCs cultured on TiO_2_ nanotubes for 7 and 14 days. Significant differences in relative gene expression between NTAPPJ with different gas sources for the same duration of exposure on TiO_2_ nanotubes are marked with ‘*’ (p<0.05). Control: untreated TiO_2_ nanotubes; NP2: TiO_2_ nanotubes with 2-min nitrogen-based NTAPPJ; NP10: TiO_2_ nanotubes with 10-min nitrogen-based NTAPPJ; AP2: TiO_2_ nanotubes with 2-min air-based NTAPPJ; AP10: TiO_2_ nanotubes with 10-min air-based NTAPPJ.

The gene expression of the specimens was measured by RT-PCR at 7 and 14 days post-irradiation. Fig. 5 shows the gene expression of ALP, Runx2, OCN and OPN. In general, the gene expression of ALP, Runx2, OCN and OPN in the NP2, NP10, AP2 and AP10 groups was higher than that of the control group (p<0.05). When comparing the effects of treatment with either nitrogen- or air-based gas on gene expression at 14 days after the same radiation dose, the nitrogen-based NTAPPJ treatment was observed to induce a higher level of gene expression than the air-based NTAPPJ treatment for a 2-min exposure time, except in the case of OCN expression (p<0.05).

## Discussion

The osseointegration of an implant is determined by the features of the implant surface. As such, the preservation of these features by prevention of material aging is important. One aim of this study was to apply the recently highlighted portable NTAPPJ to TiO_2_ nanotubes to improve their hydrophilicity and osteogenic properties while preserving the morphology of the nanotubular structure. Another aim was to circumvent the aging-related decline of these properties by taking advantage of the portability of the treatment setup.

The results of the morphological and topographical analyses before and after NTAPPJ treatment revealed that NTAPPJ treatment has no effect on the TiO_2_ nanotubular morphology or on the average roughness value (Fig. 1), which is consistent with the results of previous studies that applied NTAPPJ to the surfaces of other biomaterials [19], [32].

Despite the preservation of the morphology and topography, the hydrophilicity was significantly different between the untreated TiO_2_ nanotube surfaces and those treated with NTAPPJs. The distilled water contact angles of TiO_2_ nanotube surfaces that underwent nitrogen- or air-based NTAPPJ treatment were significantly lower than those of the control group (Table 1, *P*<0.05). This improvement in hydrophilicity is expected to contribute to the osteogenicity of TiO_2_ nanotubes [33]. Notably, the NP2 and NP10 groups resulted in contact angles of zero degrees, whereas the contact angles of the AP2 and AP10 groups were reduced compared to the control values by a smaller amount.

These changes were accompanied by changes in the chemical composition of the samples that underwent NTAPPJ treatment, as determined by XPS analysis (Fig. 2), and these modifications affected both cell attachment (Fig. 3) and cell gene expression (Fig. 5). The XPS results showed increased O-H on the surfaces of the NTAPPJ-treated TiO_2_ nanotubes compared with the controls. Additionally, the C-H and C–C peaks decreased after the nitrogen- and air-based NTAPPJ treatments, whereas the O1s and TiO_2_ peaks increased (Fig. 2).

The removal of carbohydrates in the link to the C-H peak is known to induce an increased surface energy of the biomaterial surface, resulting in an increased hydrophilic state [32]. Additionally, it is well known that carbon induces the worst effects on cell viability [34], and the reduction in carbon percentage on the surface of biomaterials is known to result in increased cellular attachment and the promotion of osteoblastic differentiation during implantation [31], [35]. In this study, the atomic percentage of each element analyzed from the XPS peaks (Fig. 2G) indicated that the carbon content in the experimental group was reduced after NTAPPJ treatment. This result indicates that the constituents of the plasma are energetic enough to break the C-C and C-H bonds at the surface layer to form radicals that yield various oxygen functionalities on the surface *via* subsequent oxidation chemistry. The resultant reduction in the carbon content of the TiO_2_ nanotube surface resulted in higher levels of hydrophilicity (Table 1), cell attachment and cell viability (Fig. 3) on the NTAPPJ-treated specimens compared with the controls. The EO gas sterilization was used as the method of sterilization for all of samples in this study, as it is commonly used for medical and dental devices. However, previous studies have indicated that cell attachment levels are lower with EO gas-treated samples, although no correlation with numbers of sterilization cycles has been presented [36], [37]. Hence, the results of reduced cell attachment for untreated TiO_2_ nanotube surfaces in our study may have been affected by EO gas sterilization. However, our previous studies with same method of sterilization, EO gas [24], [25], showed that there was no indication of compromised cellular attachment on the sterilized samples. Therefore, the effects seen here likely are not due solely to the method of sterilization used. Regarding the gas supply used for NTAPPJ treatment, the nitrogen-based NTAPPJ treatment group exhibited a lower carbon content than the air-based NTAPPJ treatment group for a given exposure time (Fig. 2G). This finding is correlated with the results of RT-PCR at 14 days of rMSC culture, which indicated that the nitrogen-based NTAPPJ treatment induced higher osteogenic gene expression than the air-based NTAPPJ treatment did [35], [38].

In general, it was demonstrated that both the nitrogen- and air-based NTAPPJ treatment resulted in an enhanced capacity for osseointegration for the TiO_2_ substrate. The application of this technology is expected to be extendable to other surface types that comprise most of the currently available Ti implants [31].

## Conclusions

In conclusion, both nitrogen- and air-based NTAPPJ treatment of TiO_2_ nanotube surfaces are effective in increasing the hydrophilicity and osseointegration of dental implants compared to untreated surfaces. However, in this study, nitrogen-based NTAPPJ treatment resulted in a higher osteogenic gene expression level and a greater decrease in the atomic percentage of carbon than those resulting from air-based NTAPPJ treatment. Hence, nitrogen gas is recommended if one gas needs to be selected; however, clinical-grade compressed air is easier to obtain (with a built-in compressor available in most clinics).

The results of this study are limited to *in vitro* applications, and *in vivo* studies or studies of other implant surfaces may be needed to confirm the validity of these results. Despite these limitations, from the results of this study, it may be concluded that NTAPPJ treatment of TiO_2_ nanotubes improve cellular attachment and differentiation without resulting in topographical changes.
